# Serum Advanced Glycation End Products as Drivers of Poor Tendon Outcomes in Diabetes? An Emerging Hypothesis and Narrative Review

**DOI:** 10.1002/edm2.70250

**Published:** 2026-05-31

**Authors:** Eric J. Gutierrez, Lauren E. Mitevski, Jacob M. Haus, Chad C. Carroll

**Affiliations:** ^1^ Department of Health and Kinesiology Purdue University West Lafayette Indiana USA; ^2^ Purdue Military Research Institute West Lafayette Indiana USA; ^3^ School of Kinesiology, University of Michigan Ann Arbor Michigan USA

**Keywords:** advanced glycation end‐products, AGE, diabetes, healing, RAGE, receptor for advanced glycation end‐products, tendon injury

## Abstract

**Introduction:**

Tendon injuries in individuals with diabetes are difficult to reverse, and the mechanisms driving poor healing, extracellular matrix disruption, and altered biomechanics remain poorly understood. Many individuals with prediabetes or diabetes are also overweight or obese, and increased visceral adiposity alters adipokine levels and promotes chronic inflammation. While multiple factors influence tendon healing and extracellular matrix (ECM) remodelling, we suggest that circulating advanced glycation end‐products (AGEs) may drive delayed healing, collagen disorganization, and compromised biomechanical properties in diabetes.

**Methods:**

A comprehensive narrative review of the literature was conducted using PubMed and Embase to synthesize current and emerging evidence on the relationship between AGEs, tendon properties, and tendon healing in diabetes. Experimental, translational, and clinical studies, as well as relevant review articles involving cell, animal, and human models, were critically analysed.

**Results:**

This narrative review explores the limited evidence linking serum AGE accumulation and Receptor for Advanced Glycation End‐Product (RAGE) activation to tendon pathology and poor healing. Traditionally, tendon dysfunction in diabetes has been attributed to non‐enzymatic collagen crosslinking. However, there is mixed evidence demonstrating increased tendon AGE content or stiffness in diabetic patients compared to age‐matched controls, suggesting alternative mechanisms. Therefore, we outline recent in vitro, animal, and human studies on AGE and RAGE in tendons and propose future directions.

**Conclusion:**

We propose that a systemic rise in serum AGEs impairs tendon healing by activating RAGE and disrupting cellular pathways required for repair. Future studies investigating the role of AGEs and other circulating factors may provide insights into the high incidence of tendon injuries and impaired healing in individuals with diabetes and related metabolic disorders.

## Graphical abstract

Tendon injuries in diabetes heal poorly, with unclear drivers of extracellular matrix disruption and altered mechanics. This narrative review synthesizes in vitro, animal, and human studies, highlighting serum advanced glycation end products as potential systemic contributors. Human evidence is limited and mixed, underscoring the need for studies integrating serum biomarkers.

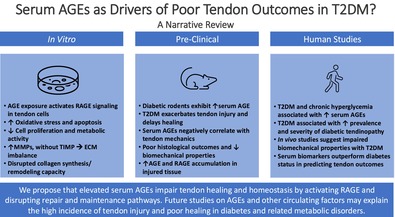

## 1 Introduction

The primary function of tendons is to translate the tension developed during muscle contraction into joint movement. Large tendons, such as the Achilles tendon, are designed to handle substantial tensile forces, enabling powerful movements like jumping or sprinting. In contrast, smaller and thinner tendons (e.g., flexor digitorum superficialis) facilitate fine, precise movements.

Beyond tensile loading, tendons also experience shear and compressive forces, particularly at the entheses and musculotendinous junctions, or during movements that occur orthogonal to the tendon's longitudinal axis. These forces are transmitted through a highly organized, hierarchical network of collagen fibres aligned with the applied load. This fibrous architecture allows tendons to withstand significant mechanical stress. However, when tendon structure deteriorates, due to injury, disease, or chronic overload, its ability to transmit force is compromised, often resulting in tendinopathy. This condition can lead to pain, dysfunction, and reduced quality of life [1, 2, 3]. While tendon injuries are common, healing is frequently incomplete, creating a cycle of re‐injury and progressive loss of function [4, 5, 6].

Tendinopathies are a significant concern for individuals with diabetes. Nearly 12% of the U.S. population is diagnosed with Type 2 Diabetes Mellitus (T2DM), with an additional 98 million Americans classified as having prediabetes [7]. Type 2 diabetes is characterized by chronic hyperglycemia, dyslipidemia, and endocrine dysregulation, all of which contribute to systemic complications. Musculoskeletal complications are also common, including accelerated osteoarthritis and a heightened risk of fractures [8]. Importantly, individuals with diabetes are nearly four times more likely to develop debilitating tendon complications compared to those without diabetes [9], particularly in the lower extremities [10]. Notably, even individuals with prediabetes (HbA1C 5.7%–6.4%) exhibit biomechanical impairments in tendons that are comparable to those observed in patients with overt diabetes [11]. These findings suggest that tendon dysfunction may begin early in the metabolic disease continuum, underscoring the need for early detection and intervention.

In contrast to those with T2DM, individuals with type I diabetes (T1D) represent approximately 5% of all diabetes diagnoses [12], although impacts to tendon health in humans are not as broadly characterized [13, 14, 15, 16]. T1D is an autoimmune disease characterized by immune‐mediated destruction of pancreatic β‐cells. It is commonly diagnosed in children and adolescents, though late onset of T1D does occur [17]. By comparison, the onset of T2DM generally develops later in adulthood and is largely associated with lifestyle factors, with additional contributions from environmental and genetic factors [18]. The pathophysiology of T2DM is characterized by insulin resistance and broad metabolic dysregulation.

Although chronic hyperglycemia is a shared feature, its aetiology, timing of onset, and underlying pathogenic mechanisms differ substantially. These differences are likely to result in divergent mechanisms and trajectories of tendon pathology. Given both the markedly higher prevalence of T2DM diagnoses and its well‐established association with diabetic tendinopathy, separate investigation of these conditions is warranted. Nonetheless, future studies that carefully examine shared and distinct mechanisms underlying tendon health in T1D and T2DM will be required for a more comprehensive understanding of diabetic tendon pathology.

Inadequate tendon healing poses a significant challenge for individuals with prediabetes and T2DM [19, 20]. Surgical intervention is often necessary to manage tendon complications in patients with T2DM [21, 22, 23]. However, diabetes compromises the regenerative capacity of tendons, leading to delayed and incomplete healing [4, 5, 6]. These deficits contribute to poorer surgical outcomes and a higher rate of post‐operative complications in patients with T2DM compared to those without diabetes [19, 20, 24].

The burden of impaired tendon healing is compounded by the increased risk of tendon injuries and hospitalizations following such injuries in people with diabetes [23, 25]. Even more troubling is the difficulty in reversing tendon damage once it occurs in this population [23, 26, 27]. Despite the clinical significance, the underlying mechanisms driving poor tendon healing, extracellular matrix disruption, and altered biomechanics remain largely unexplored. This knowledge gap highlights the urgent need for mechanistic insights to inform targeted interventions.

Hyperglycemia is believed to impair tendon healing by promoting catabolic activity and inhibiting cell proliferation, as evidenced by altered tendon properties in numerous murine models [6, 28, 29, 30, 31]. Decreased colony formation, cell death, and abnormal osteogenic and chondrogenic differentiation have also been observed within tendon stem cell populations [32, 33, 34]. However, human [25, 35, 36] and rodent [31] studies suggest that improving blood glucose control does not fully restore tendon structure and function in individuals with diabetes.

The persistence of impaired healing and poor tendon structure and function, despite normalized blood glucose levels, indicates that additional systemic factors may contribute to poor tendon outcomes. Indeed, research has shown that serum biomarkers beyond glucose, such as inflammatory mediators and serum advanced glycation end products (AGEs), may contribute to poor outcomes in people with diabetes [11, 31]. Notably, Patel et al. [11] demonstrated that various serum factors were more reliable predictors of tendon biomechanical properties than glycemic status or diabetes diagnosis alone. These findings underscore the need to look beyond glucose control and explore alternative biochemical drivers of tendon dysfunction in diabetes.

Obesity is prevalent among individuals with prediabetes and T2DM, contributing to metabolic and inflammatory complications. Increased visceral adiposity alters serum adipokine profiles and promotes chronic low‐grade inflammation, potentially negatively impacting tendon health [11]. While multiple serum factors have been linked to tendon outcomes, growing evidence from our laboratory [37, 38, 39] and others [27, 40, 41] suggests that circulating AGEs may be a key mechanistic contributor to delayed tendon healing, ECM disorganization, collagen fibril disorganization, and impaired biomechanical properties in individuals with diabetes. These findings support the hypothesis that AGEs may serve as upstream drivers of tendon degeneration, beyond the effects of hyperglycemia alone.

In this narrative review, we propose that elevated circulating serum AGE adducts trigger Receptor for Advanced Glycation End Products (RAGE) signalling. Activation of this signalling cascade may disrupt the ECM integrity, compromise tendon biomechanics, and contribute to impaired wound healing. Accordingly, the purpose of this review is to synthesize current evidence linking serum AGEs to changes in tendon integrity and healing capacity, and to identify key gaps in knowledge that warrant future investigation.

## 2 Advanced Glycation End‐Products and Tendon

### 2.1 Non‐Enzymatic Collagen Crosslinking

As collagen fibrils mature, they derive strength primarily through enzymatic crosslinking catalysed by lysyl oxidase (LOX). These enzymatic crosslinks stabilize the staggered arrangement of collagen fibrils, thereby supporting the formation of higher‐order structures that are essential for mechanical integrity. In contrast, AGEs form through a non‐enzymatic process in which free amine groups undergo covalent modification by reactive glucose or other carbonyl‐containing molecules. These modifications can lead to non‐enzymatic collagen crosslinks [42, 43, 44] that accumulate in various tissues, including muscle, bone, tendons, heart, and arteries [45, 46]. AGE crosslink formation is also positively linked with human aging [47, 48].

In tendons, AGEs typically form non‐enzymatic crosslinks at positively charged lysine and arginine residues in the telopeptide regions of collagen molecules [49, 50, 51]. Collagen‐rich tissues are particularly vulnerable to glycation due to their long half‐lives, ranging from 1 to 2 years in bone, 15 years in skin, several decades in tendons [52, 53], and up to 100 years in some cartilage [54]. Numerous in vitro studies demonstrate that non‐enzymatic crosslinking increases stiffness, elevates maximum load capacity, and reduces viscoelasticity [48, 51, 54]. Additionally, increased stiffness may impair mechanotransduction, a key process for ECM homeostasis [55], potentially diminishing the tendon's capacity to heal and remodel. However, translating in vitro findings to in vivo conditions is challenging due to differences in AGE species formed in vitro and formation timelines, hours to days in vitro versus months to years in vivo. Some evidence indicates that AGE accumulation affects post‐yield tendon mechanics without influencing modulus [51, 56]. This effect on post‐yield mechanics may be due to crosslinking interfering with molecular sliding, which influences failure properties more than the initial elastic response [57].

AGE‐modified collagen fibrils resist enzymatic degradation, thereby disrupting normal ECM remodelling [51, 56]. Ex vivo studies have shown that AGE collagen crosslinks can decrease fibril sliding and increase tissue stiffness [42, 48, 54, 56, 58, 59, 60, 61]. A key protective mechanism, discrete plasticity, is a nanoscale “kinking” phenomenon that helps tendons absorb sub‐rupture loads and is significantly hindered by AGE crosslinking. AGE crosslinks tend to form at the same telopeptide sites as enzymatic crosslinks [50], reducing or preventing kinking and making tendons more brittle [62].

Another compromised feature is strain attenuation, the tendon's ability to distribute strain along its length to protect deeper structures [63]. AGE crosslinking can impair strain attenuation function by reducing fibril sliding, leading to disproportionate strain on higher‐order structures [63]. Guatieri et al. [54] glycated rat tendon tail fascicles (RTTF) using ribose and employed various imaging modalities to observe changes at the fibril, fibre, and tissue levels during loading. At the fibril and fibre levels, loss of fibre sliding was observed concurrent with increased localized strain (i.e., reduced strain attenuation), and a monophasic response to failure was observed in glycated fascicles compared to non‐glycated controls [54].

The formation of AGE crosslinks is often associated with chronic hyperglycemia, but prolonged exposure to exogenous AGEs can also increase tissue AGE levels. In mice, exogenous AGEs accumulated in non‐weight‐bearing tendons, leading to higher initial yield points, increased elastic modulus, and an overall increase in modulus [42]. Stiffening was reduced when mice were placed on a high‐fat diet, likely due to the diet's lower AGE content, providing further evidence that exogenous AGE intake can promote subsequent tendon crosslink formation [42].

Skovgaard et al. [64] found similar levels of AGE accumulation in the energy‐storing Achilles tendon and the positional tail tendons of mice fed a standard laboratory diet (high AGE content) and a high‐fat diet (low AGE content). The authors concluded that the functional significance of the Achilles tendon may necessitate lower collagen turnover, leading to greater accumulation of AGEs over time. Thorpe et al. [52] reached a similar conclusion when they showed that the equine Superficial Digital Flexor Tendon (SDFT), a common analog for the human Achilles, had greater AGE content and reduced collagen turnover compared to low‐strain tendons. Although AGE crosslinking accumulation occurs in tendons, the extent to which it contributes to impaired tendon function in vivo, as discussed in this review, particularly in those with diabetes, remains to be determined.

## 3 Endogenous and Exogenous Sources of AGEs

### 3.1 Endogenous Formation of AGE Adducts

The production and accumulation of endogenous AGEs in vivo is a complex biochemical process involving multiple pathways, including the Maillard and Polyol pathways [65]. Despite their heterogeneity, all AGEs originate from a non‐enzymatic condensation reaction between the carbonyl group of reducing sugars (e.g., aldoses and ketoses) and a free amine group found in nucleic acids, proteins, and lipids [66]. This initial reaction produces several intermediate glycation products (e.g., HbA1C), which are easily reversible. However, through further chemical rearrangement, these intermediates become stable AGEs. AGEs are associated with hyperglycemia, and those with diabetes may have up to 7%–10% of their plasma proteins glycated, compared to less than 3% in those without diabetes [65]. AGE accumulation in both sera and tissues depends on sugar concentration and protein turnover [67]. Under normoglycemic conditions, this balance is generally favourable, although chronic hyperglycemia disrupts it, increasing the production of AGE precursors and AGEs.

Chronic hyperglycemia inhibits the glycolytic enzyme glyceraldehyde‐3‐phosphate dehydrogenase (GAPDH), leading to the accumulation of early glycolytic intermediates, such as glyceraldehyde‐3‐phosphate (GA3P) and dihydroxyacetone phosphate (DHAP) [68, 69]. These intermediates spontaneously convert to methylglyoxal, a highly reactive dicarbonyl and AGE precursor.

Importantly, this backlog of glycolytic intermediates also result in the buildup of intracellular glucose, which is then converted to fructose via the polyol pathway. Fructose may then convert to the six‐carbon AGE, 3‐deoxyglucosone, or fracture via fructolysis, generating additional reactive diacarbonyls [65]. The polyol pathway begins with aldose reductase converting glucose to sorbitol. Aldose reductase uses NADPH, which is also required for the detoxification of reactive aldehydes and the regeneration of glutathione, an important antioxidant [70]. During periods of glycolytic flux, the polyol pathway depletes NADPH, limiting its availability for antioxidant systems and impairing the reduction of reactive molecules. This cycle of cellular impairment results in intracellular protein glycation and modification, the generation of AGEs and precursors, and the diffusion of AGEs and precursors out of the cell. Intracellular AGEs can also undergo oxidative cleavage, generating additional reactive carbonyl species and promoting further protein modification and cellular impairment [71, 72, 73].

The diffusion of AGE precursors and AGEs out of the cell has two significant impacts: first, on the local cell microenvironment, and second, on the glycation of plasma proteins in systemic circulation. The AGE‐driven modification of ECM proteins is known to alter interactions between the matrix and cell, altering signalling and driving cellular dysfunction [74, 75]. Also, and of particular relevance to this work, diffusion of AGE precursors into circulation leads to the modification of plasma proteins, which accumulate in the bloodstream [76, 77, 78], and bind the AGE receptor. AGE binding induces the expression of the receptor for advanced glycation end products (RAGE), driving chronic systemic inflammation and establishing a positive feedback loop that enhances AGE accumulation, circulation, and receptor ligation.

### 3.2 Exogenous AGE Intake and Absorption

AGEs are also introduced exogenously through the diet. Foods typical of the Western diet, which are high in processed meats, fats, sugars, and soft drinks, are rich in AGEs and have been linked to AGE accumulation in both weight‐bearing and non‐weight‐bearing murine models [64].

The mechanisms of dietary AGE absorption are not fully understood. Zhao et al. [79] propose that absorption likely occurs via transcytosis, peptide transport, simple diffusion, or paracellular transport. Absorption efficiency likely depends on the molecular weight and chemical structure of the AGE compounds. Low molecular weight AGEs (< 12 kDa) may be readily absorbed, metabolized, and excreted, whereas high molecular weight AGEs (> 12 kDa) may remain unabsorbed due to limited enzymatic degradation in the gastrointestinal tract [46, 65, 80].

Accordingly, diabetic patients exhibit elevated serum AGE levels [81] along with impaired clearance of AGEs [82, 83, 84]. However, even among those without diabetes, high dietary intake of AGE‐rich foods is associated with increased AGE accumulation [47]. In both athletes and sedentary individuals, a Western diet during youth was associated with increased AGE content, as measured by skin autofluorescence, compared with those consuming a Mediterranean diet (characterized by low meat consumption, high plant intake, and inclusion of unprocessed foods) [47]. These findings suggest early‐life exposure to AGE‐rich foods may contribute to AGE accumulation during tendon development and maturation, potentially leading to long‐term complications.

Both endogenous production and exogenous intake contribute to elevated circulating AGE levels and their deposition in tissues with chronic hyperglycemia elevating the production of systemic circulating AGEs. These factors collectively increase the systemic AGE burden, enhance tissue exposure, and raise the likelihood of cellular interactions that may impair tissue function and exacerbate health conditions.

## 4 Circulating AGE Adducts and Activation of RAGE

### 4.1 Beyond Collagen Crosslinking in Tendon

Historically, AGE‐mediated crosslinking of collagen has been considered a key mechanism underlying tendon complications in individuals with diabetes [5, 54]. However, recent studies have yielded mixed findings: neither AGE content [35, 85] nor stiffness [11, 86] appears consistently elevated in patients with diabetes compared to age‐matched controls. For instance, Couppé et al. used skin autofluorescence to measure the minor AGE cross‐link pentosidine and found elevated levels in diabetic individuals compared with controls. Yet Achilles tendon biopsies from the same subject showed no significant differences in AGE content [35]. Moreover, Achilles tendon modulus did not differ between groups with poorly controlled diabetes, well‐controlled diabetes, and healthy controls. Interestingly, when diabetic subjects were pooled, and the modulus was determined at a common force (i.e., the lowest measured force among groups), diabetic subjects exhibited a significantly higher Achilles tendon modulus. However, no differences were observed at maximum force.

In contrast, Patel et al. [11] found adults with diabetes to have lower in vivo modulus compared to healthy controls when assessed using ultrasound sonography. Using amputee/cadaver tendons from individuals with diabetes (*n* = 16) and without diabetes (*n* = 6), Zellers et al. [85] found no evidence of increased collagen crosslinking compared to those without diabetes and no relationship between tendon AGE content and tensile mechanics. Although this study focused on AGE‐biomechanical relationships rather than diabetes per se, it highlights the methodological challenges in linking AGEs to impaired tendon mechanics, including tissue sample availability, variability in AGE species measurement, and differences in diabetes severity, duration, and chronological age. Animal models further complicate this picture. Diabetic mice show impaired tendon biomechanics [6] despite less evidence of tendon AGE crosslink formation than in wild‐type mice [87]. Collectively, these findings suggest that mechanisms beyond AGE crosslinking may contribute to tendon complications in vivo.

While evidence supporting cross‐link‐driven alterations in those with T2DM is inconclusive, the effect of cross‐linking cannot be dismissed. Given the well‐established structure–function relationship of proteins, non‐enzymatic modifications are expected to alter collagen structure and functional behaviour of fibrils. However, the extent to which these molecular‐level changes translate up the tendon hierarchy to influence macroscopic tendon properties remains unclear.

For example, increased levels of both enzymatic and non‐enzymatic collagen cross‐links have been reported in the patellar tendons of older men when compared to younger men, alongside age‐related reductions in collagen content, yet without any observed differences in mechanical properties [88]. These findings suggest that cross‐link accumulation alone may be insufficient to explain functional tendon changes.

Importantly, tendon alterations are strongly associated with diabetes, yet improvements in glycemic control do not completely recover structure or function in individuals with T2DM. This suggests that tendon pathology is multifactorial, may manifest differently across patients and tendon types, and likely involves pathogenic mechanisms beyond collagen cross‐linking alone.

Given the strong association between hyperglycemia, systemic AGEs formation, and tissue degeneration, we propose that circulating, particularly non‐crosslinking AGE adducts, may play a role in tendon pathology and impaired healing. Circulating AGEs can activate RAGE signalling, triggering downstream signalling pathways that may impair tendon structure and healing. This alternative hypothesis provides a complementary framework through which systemic metabolic stress, rather than matrix cross‐linking alone, may drive tendon alterations in those with T2DM.

Our perspective aligns with the “common soil theory” [89], which posits that AGE‐rich diets and increased endogenous AGEs contribute to chronically elevated serum AGE levels and tissue deposition. These elevations may foster a transition toward metabolic dysfunction and associated complications, including musculoskeletal disorders. In this framework, AGEs are both a cause and consequence of disease, a biochemical “common soil” from which systemic pathology emerges [90].

### 4.2 AGE Activation of RAGE

RAGE is a 45–55 kDa transmembrane protein belonging to the immunoglobulin superfamily. It consists of three regions: an extracellular domain, a transmembrane segment, and a cytosolic tail [91, 92, 93]. The extracellular portion includes one variable (V) domain and two constant (C) domains. The extracellular V‐C1 domains feature positively charged regions, while the C2 domain exhibits a negatively charged surface [92]. These charge distributions enable RAGE to function as a multiligand receptor, capable of interacting with both negatively and positively charged ligands.

AGEs are known to bind to the V domain of RAGE [92] where their negatively charged regions of AGE modification interact with the positively charged surface of the V‐C1 domain. Rather than promoting clearance or degeneration, this interaction initiates a prolonged period of cellular activation [91]. RAGE signalling is self‐amplifying: ligand binding activates NF‐kB, which in turn upregulates RAGE expression, creating a positive feedback loop [94].

Elevated serum AGE concentrations, as in diabetes, are presumed to increase receptor‐ligand interactions, thereby increasing reactive oxygen species (ROS) production, apoptosis, ECM degradation, and reduced cell viability [39, 58, 60]. Importantly, RAGE also binds other ligands such as S100 proteins [95] and HMGB‐1 [96], among others [97, 98], some of which have been shown to increase under hyperglycemic conditions [99, 100]. The multiligand nature of RAGE complicates the interpretation of AGE‐RAGE signalling, as multiple ligands may contribute to downstream effects. While hyperglycemia promotes AGE formation, it remains unclear whether this results in preferential AGE‐RAGE activation in tendon tissue compared to other RAGE ligands.

### 4.3 RAGE in Musculoskeletal Tissues

RAGE is expressed in a wide range of human and animal tissues, including the nervous system [101, 102], kidney [103], and the lung [104]. It is also expressed in musculoskeletal tissues, including bone [105, 106] and skeletal muscle [107]. While much of the research on RAGE has focused on its role in pathological conditions, RAGE also plays a physiological role, contributing to inflammatory responses under normoglycemic conditions [108].

We propose that chronically elevated serum AGEs and feed‐forward RAGE expression may drive tendon dysfunction in individuals with diabetes. Although RAGE expression in tendon tissue is not well characterized, emerging evidence suggests it may contribute to tendon pathology [37]. For example, a recent study by Asomugha et al. [109] found higher concentrations of localized AGE adducts in human tendinopathic posterior tibial tendons compared to non‐tendinopathic tendons. RAGE expression was confirmed in both groups, but no significant difference was observed, highlighting the need for higher‐powered human studies to clarify the role of AGE‐RAGE signalling in tendinopathy. Below, we outline the limited but growing body of evidence from in vitro, animal, and human studies examining the impact of AGEs and RAGE on tendon health.

### 4.4 AGEs, via Activation of RAGE, Impair Cellular Processes Critical for Tendon Healing and Remodelling

AGEs exert numerous harmful effects on tendon‐derived cells. These effects include increased oxidative stress and apoptosis, impaired tenogenic differentiation and proliferation of tendon stem/progenitor cells (TSPC), and cellular senescence. Patel et al. [39] demonstrated that treating tendon‐derived fibroblasts with normal‐to‐supraphysiological levels of glycated bovine serum albumin (AGE‐BSA) dose‐dependently suppressed cell proliferation under normoglycemic and hyperglycemic conditions. Mitochondrial respiration and overall metabolic activity were also diminished, suggesting that the RAGE activation compromises the energy production necessary for the proliferative and remodelling phases of tendon healing. These findings parallel observations in diabetic wound healing, where RAGE overexpression promotes fibroblast apoptosis and delays wound closure [110].

Impaired wound healing, particularly diabetic ulcers in the lower extremities, is a common complication in individuals with T2DM [111]. Wound repair in T2DM is disrupted by a multitude of interacting factors, including increased ROS production and the accumulation of AGEs, both of which have been strongly implicated in delayed and ineffective wound healing [112, 113].

Under normoglycemic conditions, RAGE expression is relatively low. However, in the diabetic wound environment, elevated AGE levels lead to sustained RAGE activation, establishing a positive feedback loop that propagates inflammation, prevents proper resolution of the inflammatory phase, and thereby impairs tissue repair [114, 115]. For example, RAGE activation of macrophages has been implicated in a sustained M1 phenotype leading to increased levels of pro‐inflammatory cytokines and growth factors (GF) [115]. While the blockage of RAGE in diabetic mice using soluble RAGE (sRAGE), a decoy receptor that scavenges circulating AGEs, resulted in rapid wound healing due to a limited inflammatory phase [116].

The accumulation of AGEs also promotes fibroblast apoptosis through numerous apoptotic pathways [117]. Fibroblasts are important cells for collagen synthesis, and a reduction in their ability to produce collagen, or promote collagen synthesis through the excretion of important GFs such as fibroblast growth factor (FGF), impairs wound healing through reduced collagen type III synthesis in early phases and type I collagen in remodelling/maturation phases [118]. These AGE‐RAGE associated impacts in diabetic wound healing likely translate to impaired tendon healing.

Supporting this concept, our laboratory has demonstrated that AGE‐BSA treatment upregulates matrix metalloproteinases (MMPs) without corresponding changes in tissue inhibitors of MMPs (TIMPs) and COL3A1 [39] resulting in an imbalance between ECM synthesis and degradation, which is also a feature of diabetic wound healing [112]. AGE‐BSA exposure also increased reactive oxygen species (ROS) production in cell cultures [39], a finding corroborated by immunofluorescence staining of rodent supraspinatus tendon cells treated with high concentrations of AGEs (500 μg/mL) [58]. Enhanced ROS production likely contributes to apoptosis and senescence, thereby reducing cell proliferation and impairing the remodelling capacity for tendon maintenance and healing.

Transcriptomic analysis via RNA sequencing of AGE‐treated tendon‐derived cells revealed altered gene expression in ECM regulation, cell survival, and connective tissue adaptation compared with vehicle‐treated cells [38]. Collectively, these in vitro findings link serum AGE levels to tendon ECM and broadly fit a pattern of reduced cellular viability and energy metabolism, disturbing the balance between collagen production and degradation.

Although few studies have investigated the effect of AGEs on TSPCs, evidence from other tissues suggests that the RAGE‐AGE interaction may mediate various harmful cellular responses, including reduced proliferation, aberrant differentiation, apoptosis, and autophagy [34, 119]. TSPCs possess multidirectional potential and play a critical role in maintaining tendon homeostasis and reversing tendinopathy. Shi et al. [26] speculate that RAGE activation in TSPCs may trigger adverse effects through the secondary activation of signalling pathways, including the Wnt/B‐catenin, p38, MAPK, Notch, ROS, and Akt/eNOS pathways.

Importantly, RAGE lacks intrinsic enzymatic activity and relies on adaptor proteins such as DIA‐1 [120] and MyD88/TIRAP [121] to initiate downstream signalling. These adaptor proteins activate pathways including MAPK [122], JAK/STAT, NF‐κB [123], and PI3K/Akt [124], which are also downstream of various cytokine receptors [125] and toll‐like receptors (TLR) [126]. Therefore, AGE‐RAGE interactions may play a critical role in hindering TSPC‐mediated tendon repair and regeneration, although further studies are needed to fully elucidate these complex cellular signalling relationships.

### 4.5 In Vivo Studies of Serum AGEs and Tendon Pathology

In rats, streptozotocin‐induced diabetes was shown to exacerbate tendon pathology in the supraspinatus tendon model [61]. Specifically, diabetic rats exhibited greater collagen fibre disruption, histological degradation (as assessed by Bonar scoring), and ECM disorganization at 2, 4, 8, and 12 weeks post‐injury compared with non‐diabetic controls. These structural impairments were accompanied by reduced biomechanical properties. Notably, serum AGE concentrations, measured by ELISA, were negatively correlated with ultimate tendon load during biomechanical testing, suggesting that elevated AGEs may contribute to tendon weakening through increased deposition or ligand‐receptor interactions.

In human studies, similar trends have emerged. In a cohort of 30 patients with rotator cuff tears, diabetic individuals were disproportionately represented in the most severe tear category (Type 3), as classified by a novel MRI‐based assessment developed by Ishitani et al. The authors used T2‐weighted, fat‐suppressed MRI images of rotator cuff stumps to classify tear severity. Immunostaining of Type 3 tears revealed elevated levels of AGE adducts and RAGE, along with increased expression of NADPH oxidase isoforms NOX‐1 and NOX‐4, key enzymes in ROS production. These tissues also showed heightened apoptosis and abnormal collagen I/III ratios, reinforcing the detrimental role of AGE‐RAGE signalling in tendon degeneration [127].

Similarly, Shinohara et al. [59] noted increased AGE accumulation in the rotator cuff tendons of diabetic patients, suggesting that AGEs contribute to reduced shoulder range of motion by increasing oxidative stress, promoting fibrosis, and altering collagen composition. These findings underscore the multifactorial impacts of AGEs on tendon tissue, including:

- ECM alterations (e.g., cross‐linking, protein adduct formation),
- Cellular dysfunction (e.g., apoptosis, oxidative stress),
- Fibrotic remodelling, and
- Biomechanical degradation.

While current human data suggest a link between serum AGEs and poor tendon outcomes, confounding variables such as age, BMI, and diabetic status complicate interpretation. Therefore, additional human studies are necessary to elucidate the mechanisms underlying impaired tendon healing. Recently, Patel et al. employed elastic net regularization that incorporated serum biomarkers, BMI, age, and diabetic status, accounting for 54% of the variability in elastic modulus in 40 adults with diabetes, pre‐diabetes, or no diabetes [11]. Notably, a simple linear model identified group differences in elastic modulus yet accounted for only 19% of the variability. These findings highlight that serum variables may be stronger predictors of tendon health than diabetic status alone. We aim to expand this work by increasing the sample size and including serum AGEs as covariates.

### 4.6 Diabetes Pharmacotherapies and Potential AGE‐RAGE Interactions

Further complicating the investigation of the effects of T2DM and elevated serum AGEs on tendons in human populations is the use of relatively novel treatments, such as glucagon‐like peptide‐1 receptor agonists (GLP‐1RAs) and sodium‐glucose cotransporter 2 inhibitors (SGLT2i), among others [128]. Notably, in the study by Patel et al. [11] over 69% of those diagnosed with T2DM were prescribed drugs to manage diabetes, cholesterol, or blood pressure. This reflects a confounding reality that must be acknowledged in any human observational or cross‐sectional study using diabetic populations.

Although use of GLP‐1R agonists or SGLT2i was not assessed in the previously mentioned study, both drugs have well‐established cardiovascular and renal benefits in those with T2DM and cardiovascular disease (CVD) and have become increasingly popular [128, 129, 130]. Recent experimental studies have also shown that these drugs may exert protective effects on neurons, kidneys, and endothelium by modulating RAGE signalling pathways [131, 132, 133]. Additionally, based on their well‐established efficacy, their clinical use has become common, with current guidelines now recommending one or both therapies for those with T2DM and other comorbidities (e.g., CVD) [129, 134]. In a T2DM cohort from our own laboratory's unpublished data, over 45% of those with T2DM are prescribed one or both of these drugs, reinforcing their common use in the general T2DM population.

GLP‐1 is an incretin hormone secreted by gastrointestinal cells in response to nutrient intake. This hormone enhances insulin secretion and exerts beneficial effects on renal and cardiac function. GLP‐1 receptors are also expressed in the central nervous system, where GLP‐1 signalling promotes satiety and reduces appetite, leading to a sustained sense of fullness [135]. Endogenous GLP‐1 is rapidly degraded by the enzyme dipeptidyl peptidase‐4 (DPP‐4), whereas GLP‐1 agonists, such as semaglutide, are structurally modified to resist DPP‐4 degradation, thereby prolonging receptor activation and systemic exposure [129, 136]. Conversely, SGLT‐2 inhibitors specifically target sodium‐glucose transporter 2 in the proximal tubular epithelium, which is responsible for the majority of renal glucose reabsorption. Inhibition of this transporter reduces renal reabsorption, promoting urinary glucose excretion, resulting in decreases in HbA1C, weight loss, and overall renal protection [129].

While both therapies demonstrate overlapping benefits, their mechanisms of action and targets are distinct, yet emerging evidence suggests that both can modulate AGE‐RAGE axis activity. For example, in vitro evidence demonstrates GLP‐1 treatment can block AGE‐induced upregulation of VCAM‐1 mRNA in human umbilical vein endothelial cells (HUVEC) via suppression of RAGE [133]. In a murine model, consistent with our findings in rat Achilles tenocytes [39], Chen et al. [131] reported that AGE exposure in mice reduced neuronal cell viability, increased apoptosis, and caused a three‐fold increase in NADPH oxidase activity. These pathological effects were significantly attenuated by twice‐daily administration of a GLP‐1 receptor agonist. Notably, AGE treatment upregulated RAGE expression via NF‐κB activation, an effect that was suppressed by GLP‐1 signalling.

Similarly, in a mouse model of T2DM, diabetic mice at 21 weeks of age exhibited elevated serum AGEs (MG‐H1), increased RAGE mRNA expression, and multiple markers of oxidative stress in the kidneys of db/db mice compared with controls [132]. Treatment with the SGLT2i, empagliflozin, for 13 weeks significantly inhibited increases in markers of diabetes (e.g., fasting glucose, HOMA‐IR, CRP), decreased serum MG‐H1, and attenuated AGE‐RAGE‐mediated oxidative stress in the kidney. In addition to reducing glomerular ECM accumulation, the SGLTi treatment attenuated RAGE‐driven inflammatory responses in the adipose tissue of diabetic mice [132].

Collectively, these findings suggest that these increasingly common therapies, despite distinct mechanisms of action, converge on shared downstream pathways, including RAGE, NAPDH oxidase, and NF‐kB. Experimental evidence from renal, neuronal, and endothelial tissues aligns closely with evidence from our laboratory implicating AGE‐RAGE driven cellular dysfunction characterized by reduced energy production, increased oxidative stress, and ECM dysregulation. Although additional studies are needed to definitively characterize GLP‐1R expression in tendinous tissues, emerging evidence suggests its presence [137, 138]. Importantly, independent of direct receptor expression on tendon cell populations, both GLP‐1Ra and SGLT2i appear capable of reducing intracellular AGE formation and subsequent release into circulation, supporting a potential indirect therapeutic benefit for those with diabetic tendinopathy.

With the increasing systemic benefits of these treatments driving their prescription in those with pre‐diabetes and T2DM, tendon outcomes of interest are likely under exposure to one or both therapies, resulting in a largely unexplored area of research in human tendon. Therefore, human studies must account for these confounding variables during experimental design. These treatments, with their demonstrated improvements to the systemic metabolomic environment, may promote improvements in the tendon microenvironment, highlighting critical unknowns such as, what are the effects of these drugs across the tendon hierarchy? And how are their benefits temporally characterized (e.g., prevention v. disease)?

### 4.7 Summary of the Evidence

In vitro studies consistently show that AGEs impair cellular function. Diabetic animal models associate elevated serum AGEs with impaired biomechanical properties and enhanced AGE‐RAGE interactions. Although human studies are limited, they suggest that chronically elevated serum AGE levels promote ECM dysregulation, reduce cellular vitality, increase apoptosis and oxidative stress, and ultimately lead to fibrosis and impaired healing.

The current evidence linking AGE‐RAGE signalling in tendons remains incomplete, leaving many questions unanswered. Future studies are required to provide direct evidence linking RAGE to impaired tendon healing and dysfunction. These studies can answer questions such as what the specific effects of RAGE ligation are in the tendon? Do fluctuating serum AGE levels have a dose–response relationship with tendon biomechanical properties and morphology? What are the effects of AGE‐RAGE ligation compared to other RAGE ligands (e.g., S100, HMGB‐1, etc.), and what is the relative importance of these ligands in tendinous tissues? Human and animal studies must build on existing experimental evidence by investigating effects of common diabetes pharmacotherapies and their interactions with the AGE‐RGE axis in musculoskeletal tissues. Finally, how applicable are current animal models, and how do AGEs influence key cellular pathways that promote growth and modulate the healing response?

## 5 Future Serum AGE Focused Studies

To establish a causal link between serum AGEs and tendon pathology, a comprehensive series of studies across the translational spectrum is necessary. High‐powered, cross‐sectional, prospective, and longitudinal human studies should be conducted in healthy, pre‐diabetic, and diabetic populations. These studies must measure key serum AGEs and correlate them with markers of tendon structure, function, and healing while also accounting for common pharmacotherapies such as GLP‐1Ra and SGLT‐2i. Longitudinal designs, similar to those used in cardiovascular risk prediction [139], may identify serum biomarkers with predictive value for tendon outcomes.

Further investigation is needed to understand differences in AGE content between serum and tendon tissue in individuals with diabetes versus those without. Particular focus should be placed on reliably quantifiable and biologically relevant AGEs, such as glucosepane and the methylglyoxal‐derived hydroimidazolone (MG‐H1) [140].

Animal studies using injury models can evaluate the therapeutic potential of AGE sequestration agents (e.g., aminoguanidine) and RAGE antagonists. These interventions should be assessed for their effects on tendon biomechanics and markers of tenocyte proliferation and differentiation during the healing process. Additionally, RAGE knockout models can offer a unique opportunity to isolate the effects of elevated serum AGEs on tendon tissue without the confounding influence of diabetes.

**FIGURE 1:**
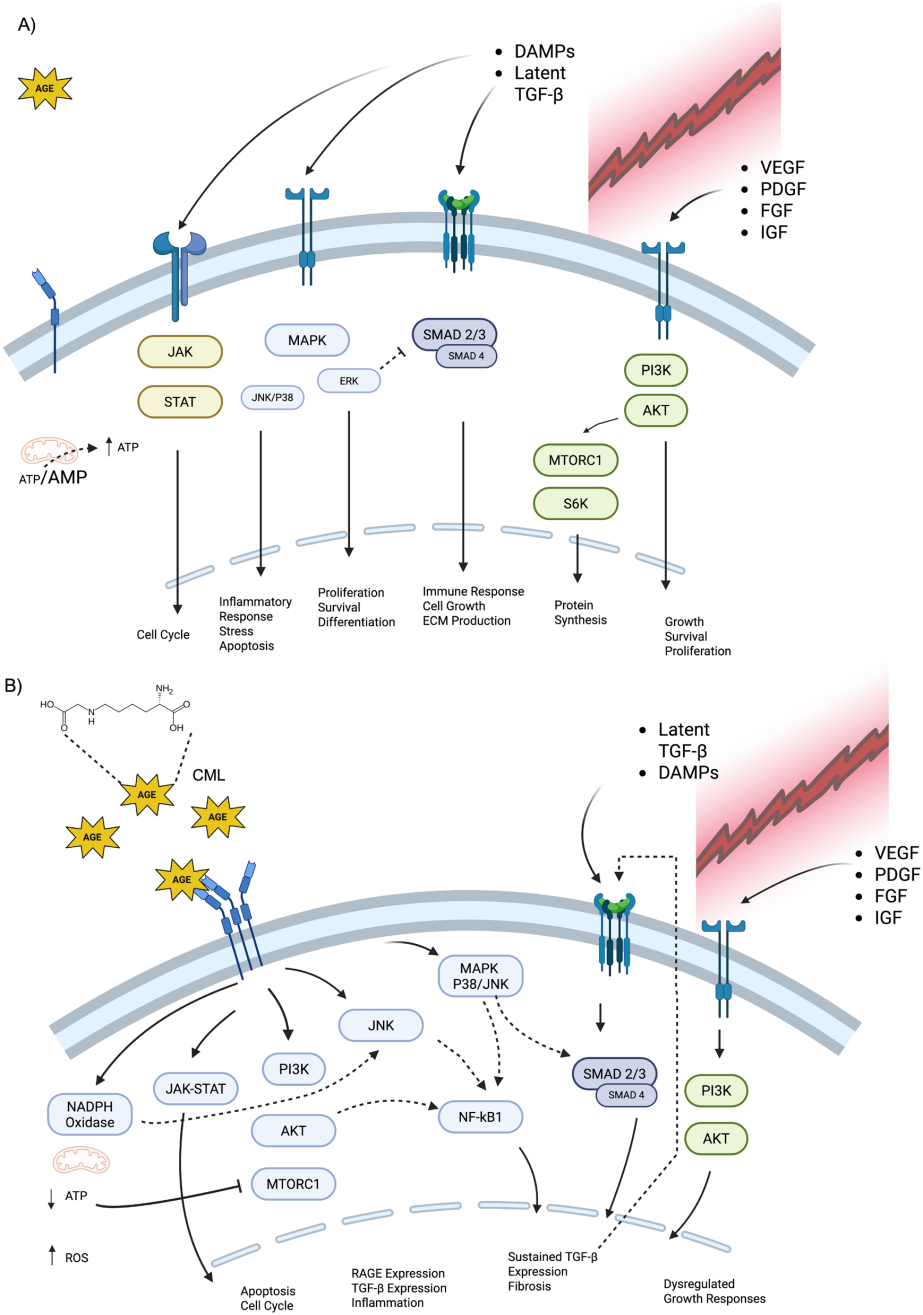
Comparison of tenocyte responses to injury. (A) Tenocyte response to injury in a low serum AGE environment. In response to tissue injury the innate and adaptive immune responses release a host of serum factors to modulate the healing process. Various growth factors and cytokines activate a cascade of cellular responses that include canonical growth and survival pathways (e.g., JAK/STAT, MAPK, TGF‐B, etc.). Over time these responses mediate the healing process. Increased metabolic demands alter the energy state and drive the production of ATP to enable anabolic responses. (B) Tenocyte Response to Injury in a High Serum AGE Environment. Similarly, the injury elicits the release of numerous serum factors. Although in a high AGE environment, RAGE ligation promotes further RAGE expression, activates numerous pathways, and creates a sustained inflammatory state through NF‐kB1 activation and TGF‐B expression. Increased oxidative stress results in a decreased energy state, an inability to promote anabolic responses, and a dysregulated healing response. Acronyms: AGE, advanced glycation end product; CML, carboxymethylysine; DAMP, damage associated molecular patterns; VEGF, vascular endothelial growth factor; PDFG, platelet derived growth factor; FGF, fibroblast growth factor; IGF, insulin‐like growth factor. Figure created with Biorender.

Tendon healing is a metabolically demanding process involving angiogenesis, neovascularization, and the influx of various serum factors to the injury site (Figure 1a). Among these, GFs play a central role in regulating cell proliferation, differentiation, and tissue remodelling. Although GF signalling is not the focus of this review, dysfunction in GF receptor signalling pathways has been implicated in diabetes‐related complications [141] and is reviewed elsewhere [142, 143, 144]. Importantly, many GF‐related signalling pathways overlap with or may be modulated by exaggerated RAGE signalling (Figure 1b). Therefore, in vitro studies using AGE‐treated tendon cells should explore how RAGE activation affects the efficacy of growth factor signalling. These studies could reveal critical interactions between AGE‐RAGE and GF pathways that influence tendon regeneration.

## 6 Conclusion

Tendons play a vital role in musculoskeletal function by transmitting muscle‐generated forces to facilitate joint movement. Despite this importance, millions of Americans with diabetes and prediabetes experience tendon pathology, functional limitations, and impaired healing, yet the mechanisms remain poorly understood. Notably, achieving clinically defined glycemic targets (HbA1C < 7) does not appear to improve tendon properties, highlighting the need to explore alternative contributors to poor outcomes.

Tendon healing is a complex phenomenon that requires coordinated metabolic activity to restore tendon function [40]. Historically, non‐enzymatic AGE crosslinking has been implicated as the primary contributor to tendon complications in individuals with diabetes [54]. However, recent studies have found limited evidence of increased crosslinking in those with diabetes compared to those without diabetes [35, 85].

Instead, elevated serum AGEs and subsequent RAGE activation have emerged as alternative mechanisms for compromised tendon properties in individuals with diabetes. Chronic RAGE ligation, triggered by elevated circulating AGE levels, may initiate a feed‐forward cycle of cellular signalling that leads to reduced cell proliferation, increased apoptosis, ECM degradation, and chronic inflammation. Sustained RAGE activation likely hinders the complex cellular crosstalk that occurs through traditional growth pathways (e.g., MAPK, PI3K, TGF‐β) during tendon remodelling and healing. Moreover, altered expression of other serum variables (e.g., growth factors, cytokines) in diabetes likely contributes to tendon complications.

Collectively, current research underscores the importance of investigating serum AGEs and related biomarkers as contributors to the high incidence of tendon injuries and poor outcomes in individuals with diabetes and metabolic disorders. Future studies should focus on:

- Delineating shared and distinct tendinopathy phenotypes between T1D and T2DM,
- Identifying the mechanisms by which serum AGEs impair tendon healing, while also accounting for the impact of matrix alterations (e.g., cross‐linking),
- Exploring the role of AGE‐RAGE signalling in tendon regeneration and its interactions with prevalent therapies,
- Evaluating the predictive value of serum biomarkers for tendon outcomes,
- And improve our understanding of tendon healing in people with diabetes, pre‐diabetes, and related metabolic conditions.

Such studies will be crucial for developing targeted therapies and enhancing surgical and rehabilitative outcomes in this rapidly growing patient population.

## Author Contributions

**Jacob M. Haus:** writing – review and editing. **Chad C. Carroll:** writing – review and editing, conceptualization. **Eric J. Gutierrez:** conceptualization, writing – original draft, methodology, writing – review and editing. **Lauren E. Mitevski:** writing – original draft.

## Funding

This work was supported by the National Institutes of Health (1R01AR081967‐01A1) to CCC. CCC received salary support from the USDA National Institute of Food and Agriculture, Hatch project 7000704.

## Conflicts of Interest

The authors declare no conflicts of interest.

## Data Availability

The authors have nothing to report.
